# The role of the *ADRB2* Thr164Ile variant in lung function determination, plasma proteome variability and other phenotypes in UK Biobank

**DOI:** 10.1183/23120541.00330-2025

**Published:** 2025-12-01

**Authors:** Katherine A. Fawcett, Robert J. Hall, Richard Packer, Kayesha Coley, Nick Shrine, Louise V. Wain, Martin D. Tobin, Ian P. Hall

**Affiliations:** 1 Division of Public Health and Epidemiology, School of Medical Sciences, University of Leicester, Leicester, UK; 2 Division of Respiratory Medicine and NIHR Nottingham Biomedical Research Centre, University of Nottingham, Nottingham, UK; 3 Leicester National Institute for Health and Care Research, Biomedical Research Centre, Glenfield Hospital, Leicester, UK

## Abstract

**Introduction:**

The effect of coding polymorphisms of the β_2_-adrenergic receptor gene (*ADRB2*) on functional properties of the receptor is well established. We recently reported a genome-wide significant association between Thr164Ile and lung function, but the contribution of this variant to other traits remains unclear.

**Methods:**

To identify pleiotropic effects of *ADRB2* Thr164Ile and other coding variants, we performed respiratory-focused and phenome-wide association studies in UK Biobank. In addition, we used available Olink proteomic data to characterise enriched pathways and upstream regulators of proteins associated with *ADRB2* polymorphisms.

**Results:**

The minor T allele of Thr164Ile was associated with reduced lung function, but not COPD or asthma or risk of exacerbations on long-acting β-agonist treatment. It was also associated with nonrespiratory traits including increased eosinophil counts and blood lipid measurements, including increased cholesterol, reduced triglycerides and reduced apolipoprotein A. Proteins associated with Thr164Ile (p≤0.01) were enriched for various pathways, with the eosinophil-raising allele associated with reduced neutrophil degranulation, immunoregulatory interactions between lymphoid and nonlymphoid cells, tumour necrosis factor binding and DAP12 interactions, as well as activation of lipid metabolism pathways, including FXR/RXR activation and LXR/RXR activation. A gene-based analysis of rare, nonsynonymous *ADRB2* variants, identified a novel association with nonrheumatic pulmonary valve disorders, but no association with lung function.

**Discussion:**

In conclusion, the lung function-lowering allele of Thr164Ile is associated with traits and proteins indicative of a role in immune and lipid metabolism pathways, suggesting potential targets for therapeutic intervention.

## Shareable abstract

**The *ADRB2* Thr164Ile variant is associated with lung function, eosinophils and blood lipid measurements, but no common respiratory conditions including COPD and asthma. Thr164Ile-associated proteins are enriched in immune and lipid metabolism pathways.** https://bit.ly/3F4KFvF

## Introduction

Three coding region polymorphisms in the *ADRB2* gene are known to alter the behaviour of the β_2_-adrenoceptor (ADRB2) in recombinant cell lines [1, 2] and in *ex vivo* human airway myofibroblasts in primary cell culture [1–3]. In brief, the Thr164Ile variant (rs1800888) alters signalling after receptor stimulation with a range of β_2_-adrenoceptor agonists, with reduced signalling for catechol ligands with the Ile164 form of the receptor [4]. The Arg16Gly (rs1042713) and Gln27Glu (rs1042714) variants alter receptor downregulation profiles following stimulation, with greater downregulation being observed with Gly16 and reduced downregulation with Glu27 [1–3].

A number of clinical studies have explored the relevance of these polymorphisms to a range of respiratory conditions, including asthma [5–7] and COPD [8–13]. Initial small studies in selected populations suggested that these polymorphisms are associated with asthma *per se*. However, the largest study to formally assess this to date, which used the UK 1958 birth cohort, showed no significant genotype-dependent effects on asthma risk [5]. Subsequently, there have been two systematic reviews addressing the contribution of these variants to asthma risk, which have come to differing conclusions [7, 14]. For COPD, a systematic review and meta-analysis of 16 studies found no association between the Thr164Ile, Arg16Gly and Gln27Glu variants and COPD, but was underpowered to investigate the Thr164Ile variant due to its lower minor allele frequency (MAF) [10]. A Danish population-based study also tested all three variants and identified associations between Thr164Ile and lung function and COPD [12]. The Arg16Gly polymorphism has also been associated with response to long-acting β-agonists (LABA) treatment in children with asthma [15, 16]. However, to our knowledge, only one study (in adults) has found an association between Thr164Ile and risk of asthma exacerbations on LABA, requiring replication [15, 17]. Interestingly, *ADRB2* polymorphisms have also been reported to be associated with nonrespiratory conditions including cardiovascular diseases [18], metabolic conditions such as obesity [19], and cancer [20], suggesting a role in multiple biological pathways and/or shared pathobiological mechanisms.

We recently described an independent, genome-wide association between the Thr164Ile variant and lung function in a multiancestry study including 588 452 individuals [21]. In contrast, the Arg16Gly and Gln27Glu variants were not associated with lung function independently of more strongly associated sentinel variants. Given that previous studies have been underpowered to study the pleiotropic effects of Thr164Ile on related respiratory and nonrespiratory conditions, we used UK Biobank to perform a phenome-wide association study. In addition, by using the exome sequencing data from the same population, we explored the potential contribution of rare coding variants to these traits across multiple genetic ancestry groups. Finally, we explored associations between the Thr164Ile variant and the plasma proteome in the subset of UK Biobank with Olink data to identify pathways underlying disease/trait associations and reveal potential opportunities for therapeutic development.

## Methods

### Study population

The UK Biobank comprises half a million volunteer participants from the UK, aged 40–69 years at recruitment. Baseline measurements, including spirometry-based lung function and anthropometric measurements, were taken at recruitment and at follow-up periods. Lifestyle and medical history information was self-reported through questionnaires and in a nurse-led interview. Secondary care records (hospital episode statistics) are also available, and 45% of participants have linked primary health care records, including prescription records. Blood samples were taken at baseline for genome-wide genotyping and were later used for exome and whole-genome sequencing. Protein measurements for 2923 proteins using the Olink platform were also recently released for 54 219 participants (refer to [22] for details of data processing and quality control).

Genotyping was carried out using the Affymetrix Axiom UK BiLEVE array or the Affymetrix Axiom UK Biobank array. These data were used as a basis for assigning individuals to ancestry groups using ADMIXTURE v1.3.0 [21] and for excluding related individuals. The Thr164Ile, Arg16Gly and Gln27Glu polymorphisms were directly genotyped in UK Biobank. Genotypes for rare variants in *ADRB2* were extracted from exome sequencing data. Exome capture was performed using the IDT xGen Exome Research Panel v1.0 including supplemental probes, and paired-end sequenced using the Illumina NovaSeq 6000 platform [23]. Variants were called using DeepVariant 0.0.10 and aggregated into genomic variant call format (gVCF) files with GLnexus 1.2.6 using the default joint-genotyping parameters for DeepVariant.

### Phenome-wide association studies

We used DeepPheWAS v0.2.0 [24] to test association between *ADRB2* coding variants and up to 1908 clinically relevant traits in UK Biobank participants of European ancestry (supplementary figure S1, supplementary table S1). Briefly, this software constructs phenotype tables comprising inverse normal transformed continuous traits measured at UK Biobank assessment centres or from primary care records, as well as binary traits based on self-report and/or diagnostic codes within linked healthcare records (see supplementary table S2 for sample sizes for each trait). We also excluded related individuals (first cousins or closer), preferentially keeping those with lower missingness (for post-filtered sample sizes; supplementary table S3). Binary traits with <50 cases and quantitative traits measured in <100 individuals were excluded. Association testing was then performed using either linear regression (for quantitative traits) or logistic regression (for binary traits), adjusting for age, sex, genotyping array and the first 10 ancestry-based principal components (PCs). The false discovery rate was set to 1%. Power calculations revealed that, for Thr164Ile, we have >99% power to detect the previous effect size on forced expiratory volume in 1 s (FEV_1_)/forced vital capacity (FVC), >99% power to detect the previously reported association with COPD (OR 1.46) [12], and >80% power to detect OR ≥1.17 for asthma.

In order to test for association with rare *ADRB2* variants, gene-based tests burden tests were run using the exome sequencing data. Regenie v3.3 was used to create masks of predicted loss of function and missense variants within five different frequency categories: singleton variants, variants with MAF<0.00001, MAF<0.0001, MAF<0.001 and MAF<0.01. Variants with a read depth of <10 in >10% of samples were excluded. Lists of variants included in each mask are given in supplementary table S4. Individuals were coded as 0 if they had no rare alleles across the *ADRB2* gene; 1 if they were heterozygous for at least one rare allele; and 2 if they were homozygous for at least one rare allele. For both single-variant and gene-based analyses, summary statistics were output to tables and results visualised in graphs using DeepPheWAS.

### Respiratory-focused phenome-wide association studies

We conducted a look-up of the Thr164Ile variant in 28 genome-wide association studies (GWAS) across 11 clinical respiratory traits: asthma, bronchiectasis, bronchopneumonia, chronic bronchitis, COPD, chronic sputum production, emphysema, idiopathic pulmonary fibrosis, respiratory infections and interstitial lung abnormalities and pneumothorax [23–39]. Some of the GWAS summary statistics are publicly available (www.ebi.ac.uk/gwas/) and some are available upon request (supplementary table S5).

### Smoking subgroup analyses

We tested associations with COPD and asthma in smoking subgroups using logistic regression models in never-smokers and ever-smokers (based on UK Biobank field 20160). For asthma, we included age, sex, genotyping array and ancestry-based PCs in the model, and for COPD, we also included standing height as a covariate.

### Asthma exacerbations and LABAs

We tested for association between Thr164Ile and asthma exacerbations in individuals with asthma who self-reported taking LABAs. Asthma exacerbations were identified from primary and secondary care records, as described previously [28]. Briefly, individuals with asthma were identified as those self-reporting asthma or with a diagnostic code for asthma in primary or secondary care records. Individuals with chronic bronchitis, emphysema or COPD (evidenced by self-report or diagnostic codes in healthcare records or, in the case of COPD, FEV_1_/FVC ratio <0.7) were excluded. Exacerbations were defined using the linked healthcare records as any of 1) hospitalisation with a primary diagnosis of asthma; 2) hospitalisation with a secondary diagnosis of asthma and a primary diagnosis of respiratory infection or an asthma-associated condition; 3) hospitalisation with a secondary diagnosis of asthma and a primary diagnosis of chest pain/dyspnoea, as long as the individual had no record of a cardiac condition; 4) diagnostic codes for exacerbations in primary care diagnostic records; or 5) oral corticosteroid bursts in primary care prescription records. Individuals taking LABAs were identified from self-reported medication data (codes provided in supplementary table S6). Individuals who did not self-report taking LABAs, but have LABAs in primary care prescriptions records, were excluded from the analysis, as these individuals may have taken LABAs for only a short period of time or been prescribed LABAs but not used them.

Among individuals with asthma who were taking LABAs (n=3251), the association between exacerbation status (having had at least one exacerbation compared to having had no exacerbations) and Thr164Ile was tested using a logistic regression model, adjusting for sex, age, age^2^, ever-smoking and the first 10 genotyping-based PCs. Power calculations indicated that we would have 80% power to detect OR ≥1.78.

### Multiancestry analyses

Given the lower frequency of the Thr164Ile variant in non-European ancestry clusters (MAF 0.002, 0.000 and 0.005 in the African, East Asian and South Asian clusters, respectively), we were underpowered to detect its effects on lung function in these groups (power 10%, 0% and 18%, respectively). However, we extended our gene-based phenome-wide association study to investigate predicted damaging rare variants in individuals of African, East Asian and South Asian ancestry. As for European ancestry individuals, Regenie v3.3 was used to create masks of predicted loss of function and missense variants within five different frequency categories: singleton variants, variants with MAF <0.00001, <0.0001, <0.001 and <0.01. Variants with a read depth of <10 in >10% of samples were excluded. Individuals were coded as 0 if they had no rare alleles across the *ADRB2* gene; 1 if they were heterozygous for at least one rare allele; and 2 if they were homozygous for at least one rare allele. Sample sizes for each trait before and after removal of related individuals are given in supplementary tables S2 and S3, respectively. Lists of variants included in each mask are given in supplementary table S4. Due to the small sample size and the frequency of the variants, we were underpowered to detect effect sizes typical of complex diseases and traits and it should be noted that lung function, COPD and asthma were excluded from these analyses by DeepPheWAS due to low sample size. Quanto v1.2.4 was used for power calculations.

### Protein analysis

Protein levels were measured from blood using the Olink platform in 54 219 UK Biobank participants. The latest release of these data includes 2923 unique protein assays across two tranches, not including ADRB2. We tested for association between Thr164Ile and untransformed log_2_ fold protein levels in individuals of European ancestry with Olink data for tranches 1 and 2 (supplementary table S1). We tested an additive genetic model and included covariates: Olink batch, age, sex, genotyping array and the first 10 genotyping-based PCs. All proteins showing association (p<0.01) were used as input to Qiagen Ingenuity Pathway Analysis (IPA) v24.0.1, along with their p-value and β-value, to identify enriched pathways and potential upstream regulators. Colocalisation was carried out using coloc.susie software v5 [40] to ascertain whether both the Thr164Ile lung function signal and the associated protein signals were driven by the same variant, with regional summary statistics from lung function and protein analyses and a linkage disequilibrium reference panel based on 10 000 randomly selected European individuals from UK Biobank. All variants within a 2-Mb region centred on the Thr164Ile variant and with a MAF >0.001 were included in this analysis. We extended these analyses to Arg16Gly and Gln27Glu to ascertain overlap between proteins and pathways impacted by *ADRB2* coding polymorphism.

### Code availability

Documentation and code for DeepPheWAS is available at https://github.com/Richard-Packer/DeepPheWAS

Code for proteomics analysis across UK Biobank imputed genotype data is available at https://github.com/legenepi/ukb_olink_pqtl

## Results

### Phenome-wide association studies

**FIGURE 1:**
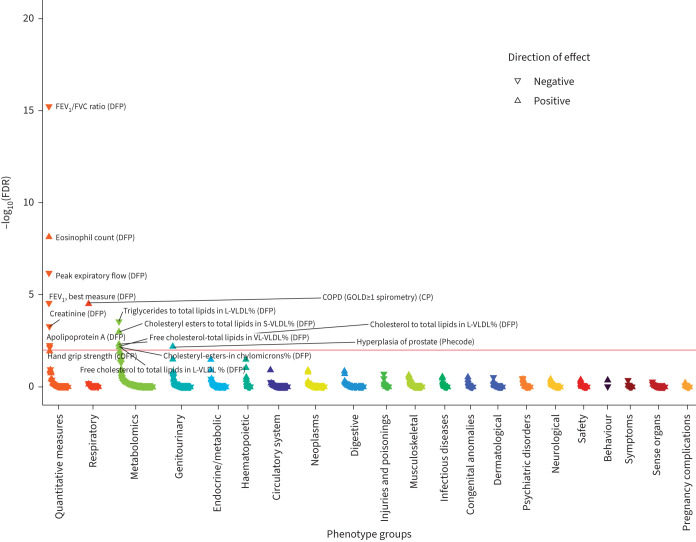
Phenome-wide association of 1746 UK Biobank traits with the β_2_-adrenergic receptor gene (*ADRB2*) Thr164Ile variant. Each triangle is a trait generated by DeepPheWAS software v0.2.0 and colour-coded according to phenotypic category. The −log_10_ of the false discovery rate (FDR) is generated from association testing: linear regression (for quantitative traits) or logistic regression (for binary traits), adjusting for age, sex, genotyping array and the first 10 ancestry-based principal components. The red line indicates the FDR threshold of 1%. The directions of effects are aligned with the minor T allele. FEV_1_: forced expiratory volume in 1 s; FVC: forced vital capacity; DFP: data field phenotype; cDFP: combined DFP; CP: composite phenotype; VLDL: very low-density lipoprotein; L-VLDL: large VLDL; S-VLDL: small VLDL; VL-VLDL: very large VLDL; GOLD: Global Initiative for Chronic Obstructive Lung Disease.

Given the genome-wide significant association we described previously between the *ADRB2* Thr164Ile variant [21] and lung function, we performed a phenome-wide association study (PheWAS) in UK Biobank in order to understand its clinical relevance across a broad range of respiratory and nonrespiratory traits. As previously shown, the minor T allele was associated with reduced lung function, including FEV_1_/FVC (p=3.06×10^−19^) and peak expiratory flow (p=9.95×10^−10^). It was also associated with increased eosinophils, a variety of blood lipid measurements including increased cholesterol, reduced triglycerides and reduced apolipoprotein A, as well as reduced creatinine and hand grip strength. The only binary outcomes associated with Thr164Ile were spirometrically defined COPD (Global Initiative for Chronic Obstructive Lung Disease stage ≥1) and hyperplasia of the prostate (figure 1, full results in supplementary table S7). There was no significant association with COPD defined using self-report and diagnostic codes in electronic healthcare records in UK Biobank (OR 1.01, 95% CI 0.93–1.09; p=0.873) or in a look-up of the Thr164Ile in 28 GWAS of respiratory diseases (supplementary figure S2 and supplementary table S8).

Due to previous reports that the effects of *ADRB2* coding variants can be modified by smoking status [13, 41–43], we also tested association of Thr164Ile with self-reported and electronic healthcare record-coded COPD and asthma in UK Biobank never- and ever-smokers separately. No associations were found within these subgroups (supplementary table S9) and there was no evidence for an interactive effect between Thr164Ile and smoking on either COPD (p=0.958) or asthma (p=0.633).

Additionally, some prior studies have identified associations between *ADRB2*-coding single nucleotide polymorphisms (SNPs) and COPD and asthma under different genetic models [7, 13]. We therefore tested Thr164Ile under a dominant and recessive model in UK Biobank but found no associations with either COPD or asthma (supplementary table S10), although given the low frequency of the Thr164Ile variant, these studies are likely to be underpowered to detect a small effect.

The previously reported association between Thr164Ile and exacerbation risk on LABA treatment was tested in 3251 UK Biobank individuals with asthma who had self-reported taking LABAs at recruitment. Of these 3251 individuals, 2276 had experienced at least one exacerbation identified from primary and secondary care records. We found no association between Thr164Ile and risk of being an exacerbator (OR 1.146, 95% CI 0.733–1.847; p=0.561).

### Proteins and pathways

For mechanistic insight into the effects of Thr164Ile on lung function, we tested its association with 2923 proteins measured in UK Biobank blood samples (n=54 219) using the Olink platform. Only three associations were discovered at a p-value threshold corrected for the effective number of independent tests (as calculated by the MeffLi method [44]), which was with MEGF10 (multiple epidermal growth factor-like domains protein 10) (p=7.84×10^−7^), tumour necrosis factor (TNF) superfamily member 13b (TNFSF13B) (p=3.05×10^−5^) and T-cell immunoglobulin and mucin domain containing 4 (TIMD4) (p=3.27×10^−5^).

**FIGURE 2:**
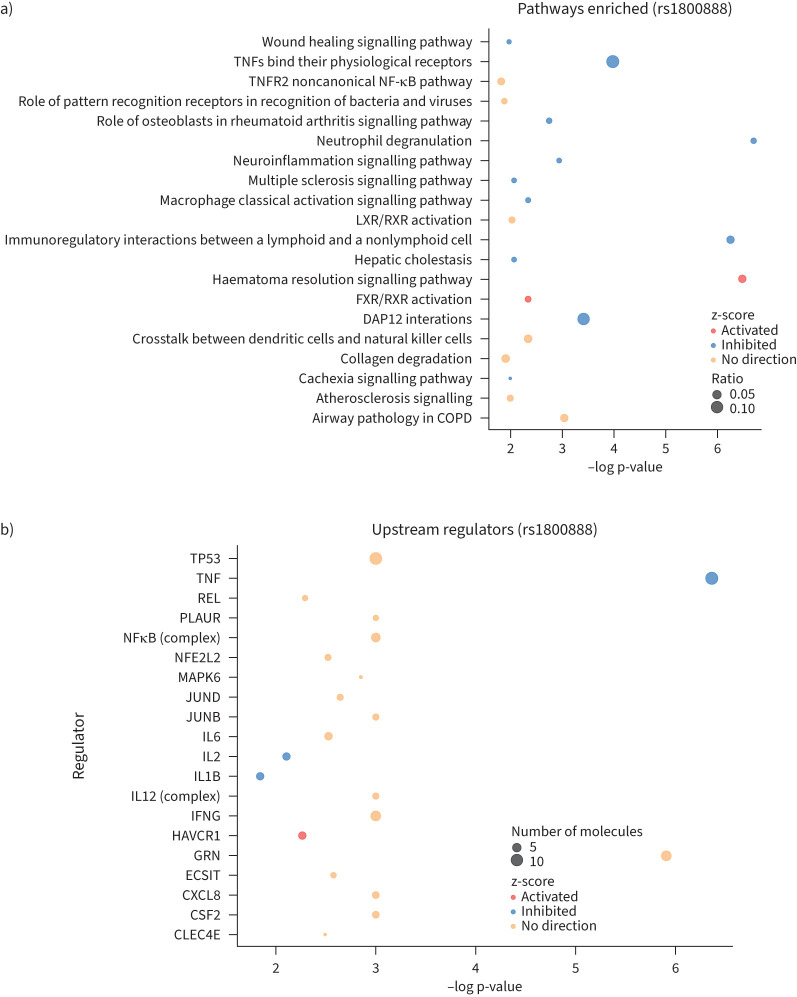
Bubble plots of enriched pathways and upstream regulators among proteins associated with the Thr164Ile variant, generated using Ingenuity Pathway Analysis. The colour of the circles indicates whether or not the pathway is predicted to be activated or inhibited (or whether no direction can be determined), and the size of the circles indicates the percentages of proteins in the full pathway that are among the Thr164Ile-associated proteins. TNF: tumour necrosis factor; TNFR: tumour necrosis factor receptor; IL: interleukin; HAVCR: hepatitis A virus cellular receptor.

At a nominal significance threshold (p<0.01), the Thr164Ile variant was associated with 61 proteins (supplementary table S11). These proteins were analysed in IPA to identify enriched canonical pathways and potential upstream regulators (figure 2, supplementary tables S12 and S13). 37 pathways were enriched among proteins associated with the Thr164Ile minor T allele (supplementary table S12). These included inflammatory response pathways such as neutrophil degranulation, immunoregulatory interactions between a lymphoid and a nonlymphoid cell, and TNFs binding to their receptors (predicted to be inhibited as a result of Thr164Ile-associated protein changes). There was also enrichment for some metabolic pathways, including FXR/RXR activation, LXR/RXR activation, hepatic cholestasis (inhibited), which are all involved in lipid metabolism. Finally, pathways associated with wound healing were also enriched, including hematoma resolution (activated). Predicted upstream regulators (supplementary table S13) were dominated by those involved in immune response including TNF, interleukin (IL)-2 and IL-1β, which were predicted to be inhibited upstream regulators, while hepatitis A virus cellular receptor 1 (HAVCR1) was predicted to be activated.

### Rare coding variants in *ADRB2*

**FIGURE 3:**
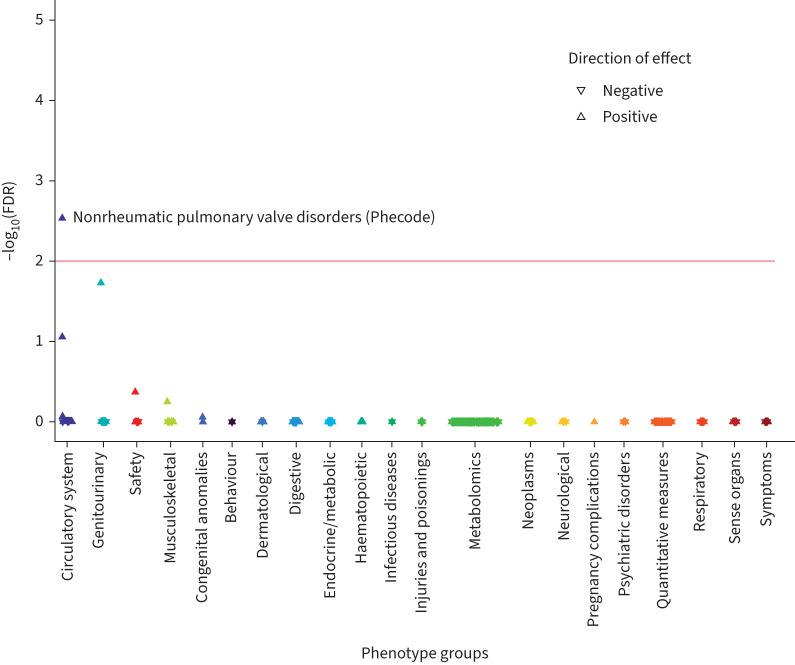
Phenome-wide association of UK Biobank traits with a gene-based model of rare (minor allele frequency <0.0001), nonsynonymous β_2_-adrenergic receptor gene (*ADRB2*) variants. Each triangle is a trait generated by DeepPheWAS software v0.2.0 and colour-coded according to phenotypic category. The −log_10_ of the false discovery rate (FDR) is generated from association testing: linear regression (for quantitative traits) or logistic regression (for binary traits), adjusting for age, sex, genotyping array and the first 10 ancestry-based principal components. The red line indicates the FDR threshold of 1%.

Exome sequencing across the full UK Biobank cohort detected 264 rare (MAF <0.01) nonsynonymous changes in *ADRB2* (supplementary table S14). To assess whether the effects of Thr164Ile on lung function might be mediated by rare variants or whether rare variants had independent effects on respiratory or non-respiratory traits in UK Biobank, we performed another PheWAS in individuals of European genetic ancestry. Given lack of statistical power to detect effects of single, rare variants, we collapsed these variants together using different maximum MAF cut-offs. Only one of these models (variants with MAF <0.0001) showed any associations at an FDR <0.01: namely with nonrheumatic pulmonary valve disorders (OR 11.497, 95% CI 4.247–31.121; p=1.53×10^−6^; figure 3 and supplementary table S15).

In UK Biobank participants within African, East Asian and South Asian genotyping-based clusters, there were no associations in *ADRB2* gene-based tests, even at a lenient FDR threshold of 5% (supplementary tables S16–S18).

### Arg16Gly and Gln27Glu variants

The two other coding polymorphisms in *ADRB2* (Arg16Gly and Gln27Glu) have minor allele frequencies of 0.42 and 0.32, respectively, in UK Biobank. They are in linkage disequilibrium (r^2^=0.43); the minor A allele of Arg16Gly tending to be inherited with the major C allele of Gln27Glu. In previous studies, they have shown inconsistent associations with a variety of respiratory conditions and associated traits. We therefore performed PheWAS of these SNPs and showed association with increased eosinophil count and decreased neutrophil count in the blood (supplementary figure S2A and B, full results in supplementary tables S19 and S20). They showed no association with respiratory conditions (supplementary table S8), including COPD or asthma, in UK Biobank ever- and never-smokers and under different genetic models (supplementary tables S9 and S10, respectively).

In the UK Biobank Olink data, the Arg16Gly and Gln27Glu variants were associated with 11 and 22 proteins, respectively, at an adjusted p-value threshold (or −log_10_p=4.47, highlighted red in supplementary tables S21 and S22) and with 76 and 123 proteins, respectively, at a nominal p-value threshold (p<0.01). We used IPA to identify enriched canonical pathways and potential upstream regulators (supplementary tables S23–S26, supplementary figure S4). The results shown are aligned to the allele associated with higher eosinophil and lower neutrophil numbers. In brief, Arg16Gly-associated proteins were enriched in 17 pathways (Benjamini–Hochberg corrected p<0.05) including neutrophil degranulation, wound healing, extracellular matrix degradation, osteoblast function in rheumatoid arthritis (all predicted to be inhibited), and matrix metalloproteinase (MMP) activation and osteoarthritis (no directionality predicted). Gln27Glu-associated proteins were enriched in 51 pathways, including 12 that overlap with the Arg16Gly-enriched pathways as well as inflammatory pathways such as IL-17 signalling, type 1 and 2 T-helper (Th1 and Th2) pathways, pathogen-induced cytokine storm pathway (all predicted to be inhibited), and airway inflammation in asthma (no specific directionality predicted). We compared these results to the pathways enriched among Thr164Ile-associated proteins and found five overlapping pathways between all three coding variants (supplementary figure S5A): neutrophil degranulation (inhibited), wound healing signalling pathway (inhibited), activation of MMPs (no direction), airway pathology in COPD and role of osteoblasts in rheumatoid arthritis signalling pathway (inhibited). Analyses of upstream regulators in general showed no clear directional effects, although for Arg16Gly TNF and IL-17A were both predicted to be inhibited upstream regulators, while for Gln27Glu predicted inhibited upstream regulators included TNF, IL-18 and c-Jun NH2-terminal kinase (JNK). Overlap of upstream regulators between all three variants is shown in supplementary figure S5B.

## Discussion

Here, in comprehensive PheWAS analyses, we confirm that the Thr164Ile *ADRB2* variant is associated at a genome-wide significant level with lung function in UK Biobank, and in addition show the minor T allele is associated with a range of nonrespiratory phenotypes including eosinophil counts and blood lipid measurements as well as reduced creatinine and hand grip strength. Rare variant analyses, using a collapsing gene-based approach, identified an association with nonrheumatic pulmonary valve disorders. However, neither rare *ADRB2* variants nor the common *ADRB2* codon 16 and 27 polymorphisms showed association with lung function, COPD, asthma or any other respiratory diseases.

Previous smaller association studies exploring *ADRB2* coding region polymorphisms and asthma risk have yielded inconsistent results, exemplified by two systematic reviews [7, 14]; one concluding that Arg16Gly and Gln27Glu polymorphisms are associated with asthma risk, while the other found no evidence for association. Our study provides a definitive answer to this issue. The association of Thr164Ile with reduced lung function in UK Biobank is consistent with the results of a smaller Danish population-based study [12], which found association between Thr164Ile and reduced lung function and increased risk of COPD.

ADRB2 signals predominantly through elevation of intracellular cyclic AMP and protein kinase A activation. ADRB2 stimulation is known to cause many downstream effects including altered gene expression in a wide range of cell types driven through cAMP response element binding protein (CREB) family members binding to CRE regulatory sites (reviewed in [45]). Given the PheWAS associations we observed between the Thr164Ile variant and lung function, eosinophil count and lipid measurements, we explored potential underlying mechanisms using the Olink plasma proteome data in a subset of UK Biobank. Among 61 proteins nominally associated with Thr164Ile, there were a number of enriched pathways and upstream regulators. Interestingly, the eosinophil-increasing allele was associated with protein changes indicative of inhibition of immune pathways including neutrophil degranulation, immunoregulatory interactions between lymphoid and nonlymphoid cells, TNF binding and DAP12 interactions among others. Consistent with its association with blood lipid measurements, it was also associated with pathways relevant to lipid metabolism including FXR/RXR activation, LXR/RXR activation and hepatic cholestasis. Interestingly, both exogenous and endogenous lipids are known to play a role in immune regulation, including immune cell activation, differentiation and expansion, suggesting possible cross-talk between these pathways and a role in immune-related traits and diseases [46].

Probably on account of its low frequency, only three proteins were associated with Thr164Ile at a p-value threshold adjusted for multiple testing (MEGF10, TNFSF13B and TIMD4). Although speculative, the association of the lung function-lowering allele with lower MEGF10 levels suggests that this protein could have a role in maintaining airway calibre. MEGF10 is a member of the multiple epidermal growth factor-like domains protein family, and plays a role in cell adhesion, motility and proliferation. It is also an essential factor in the regulation of myogenesis where it is believed to control the balance between skeletal muscle satellite cell proliferation and differentiation through regulation of the notch signalling pathway. Mutations in *MEGF10* are associated with myopathies, and so it is possible that lower MEGF10 alters airway wall morphology through effects on airway myofibroblasts leading to the observed FEV_1_/FVC signal in our study [47]. In a look-up of in-house spatial transcriptomic data using fixed human lung tissue, *MEGF10* transcripts were detected in airway smooth muscle *in situ* (data not shown). Interestingly, myopathy caused by *MEGF10* mutation in a range of nonhuman models can be reversed by selective serotonin reuptake inhibitors [48], so if reduction in MEGF10 function is causally related to lung function there is a possible route to drug repurposing.

An alternative explanation is that this protein quantitative trait loci association could have arisen due to an indirect effect of Thr164Ile on regulation of MEGF10. For example, because Thr164Ile is also associated with eosinophil count, proteins expressed in eosinophils could underlie the observed association. However, according to the Human Protein Atlas (www.proteinatlas.org/), MEGF10 is not detected in immune blood cells. A search of the STRING, IntAct, BioGRID and the Molecular INTeraction databases revealed no known interactions between ADRB2 and MEGF10.

The other two proteins associated with Thr164Ile, TNFS13B and TIMD4, have known roles in immune regulation. TNFSF13B is a cytokine with a role in proliferation, differentiation and activation of B-cells and polymorphisms in this gene have been associated with autoimmune conditions [49]. TIMD4 is a phosphatidylserine receptor with roles in clearance of apoptotic cells and T-cell regulation, including promotion of type 2 inflammation [50]. It should be noted that we were unable to test the association between Thr164Ile and levels of ADRB2 as this protein was not included in the UK Biobank Olink data. As ADRB2 is a membrane-bound G-protein coupled receptor, we would not expect to find it in blood serum, consistent with its absence from blood in the Human Protein Atlas.

One interesting observation from our results is the dissociation of the genetic association signals for eosinophilia and asthma risk at the *ADRB2* locus. Previous studies have generally shown substantial overlap between the genetic associations for these two phenotypes [51], and there is considerable evidence that eosinophilia is associated with poorly controlled asthma and treatment response in COPD [52, 53]. The dissociation we observed between eosinophil and asthma phenotypes implies that not all genetic factors involved in control of eosinophil levels lead inevitably to asthma.

This study had several limitations. Firstly, we only investigated nonsynonymous coding region variants, rather than synonymous variants, or intronic or upstream/downstream variants that might have roles in regulation of *ADRB2* splicing or expression. Secondly, our analysis of the Thr164Ile variant under a recessive genetic model may have been underpowered due to there being only 107 individuals of European ancestry carrying two copies of the minor T allele in UK Biobank. Thirdly, most of our analyses were limited to the largest ancestry group in UK Biobank, namely white British individuals. A multiancestry analysis of *ADRB2* variants in additional, diverse cohorts could test generalisability of these findings to other world populations. Finally, our analysis of Thr164Ile and exacerbation risk in asthmatic individuals taking LABAs should be interpreted cautiously, as the temporal relationship between treatment and exacerbation was difficult to assess. Furthermore, LABAs were almost always being taken alongside inhaled corticosteroids so independent effects of LABAs cannot be determined.

Taken together these results show that it is the Thr164Ile variant of *ADRB2* drives the lung function genetic association signal at this locus potentially through alteration of immune- and lipid-metabolism pathways. We identified a number of enriched biological pathways among Thr164Ile-associated proteins and potential target proteins for therapeutic intervention. We also show that *ADRB2* coding variants are unlikely to be clinically important for asthma risk or for response to LABAs in adults.

## Supplementary material

**Please note:** supplementary material is not edited by the Editorial Office, and is uploaded as it has been supplied by the author. Supplementary figures 00330-2025.SUPPLEMENT Supplementary tables 00330-2025.SUPPLEMENT
