# Supplementary material for: The role of the ADRB2 Thr164Ile variant in lung function determination, plasma proteome variability and other phenotypes in UK Biobank
Source: ERJ Open Res. 2025 Dec 1;11(6):00330-2025. doi: 10.1183/23120541.00330-2025 (PMC12683596; doi:10.1183/23120541.00330-2025)
Supplement: Supplementary file 1 [file 00330-2025.SUPPLEMENT.pdf]

Supplementary Figures

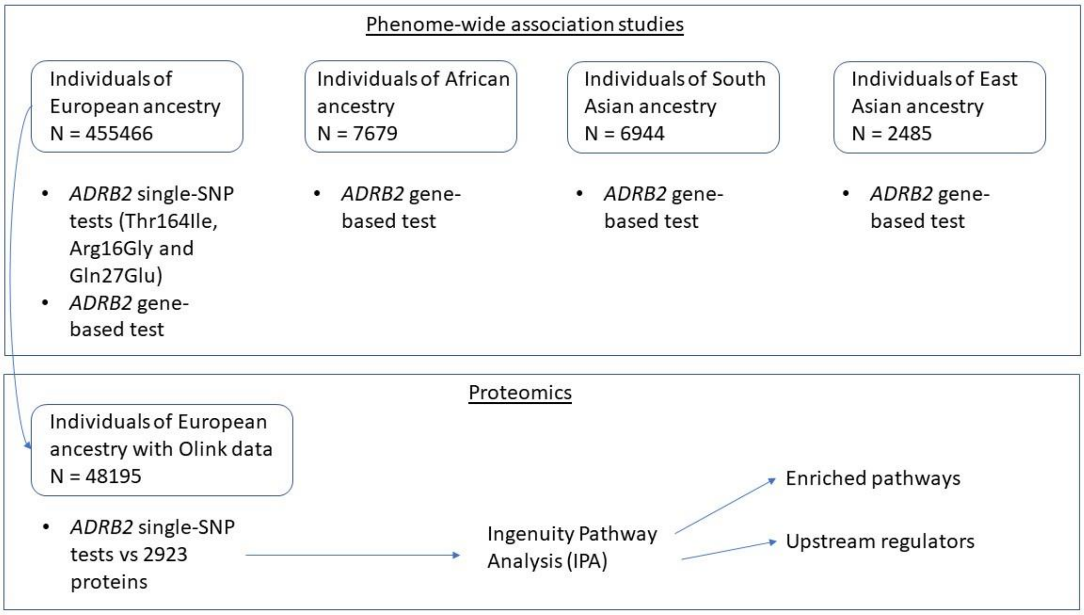

Supplementary Figure 1. Study design for phenome-wide association studies and proteomics study in UK Biobank participants.

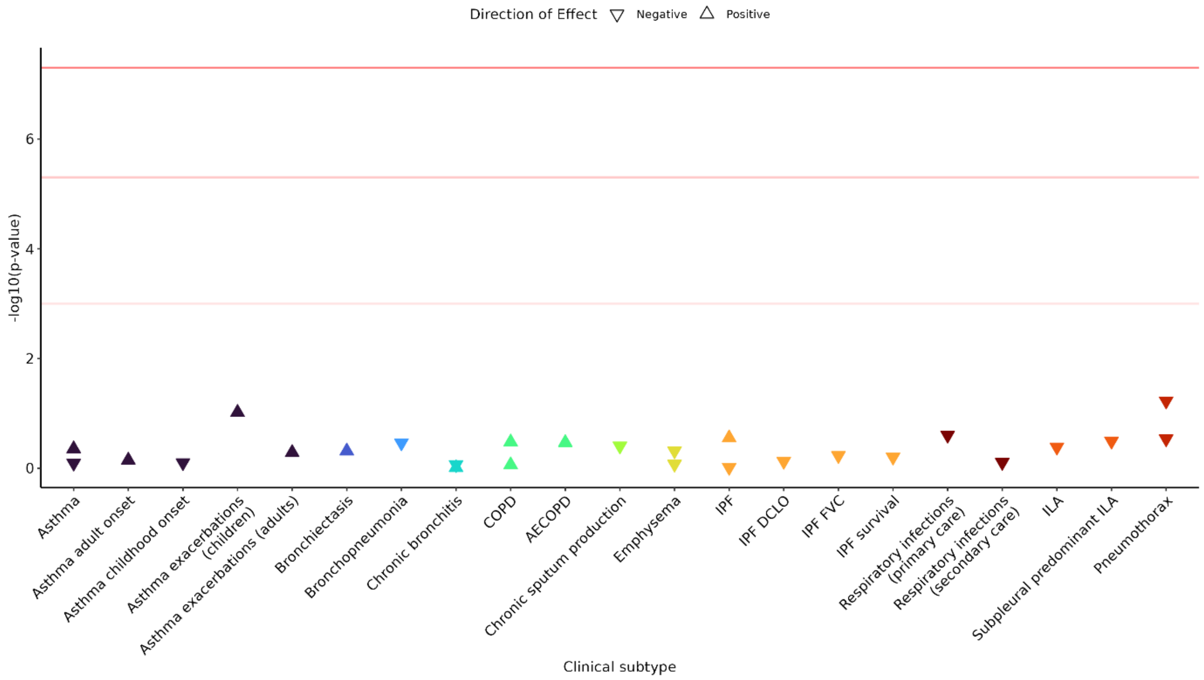

Supplementary Figure 2. Association of Thr164Ile variant (rs1800888) with respiratory diseases based on publicly available and in-house genome-wide association studies. Top to bottom, the three red lines represent the p value thresholds 5×10^{-8}, 5×10^{-6} and 1×10^{-3}.

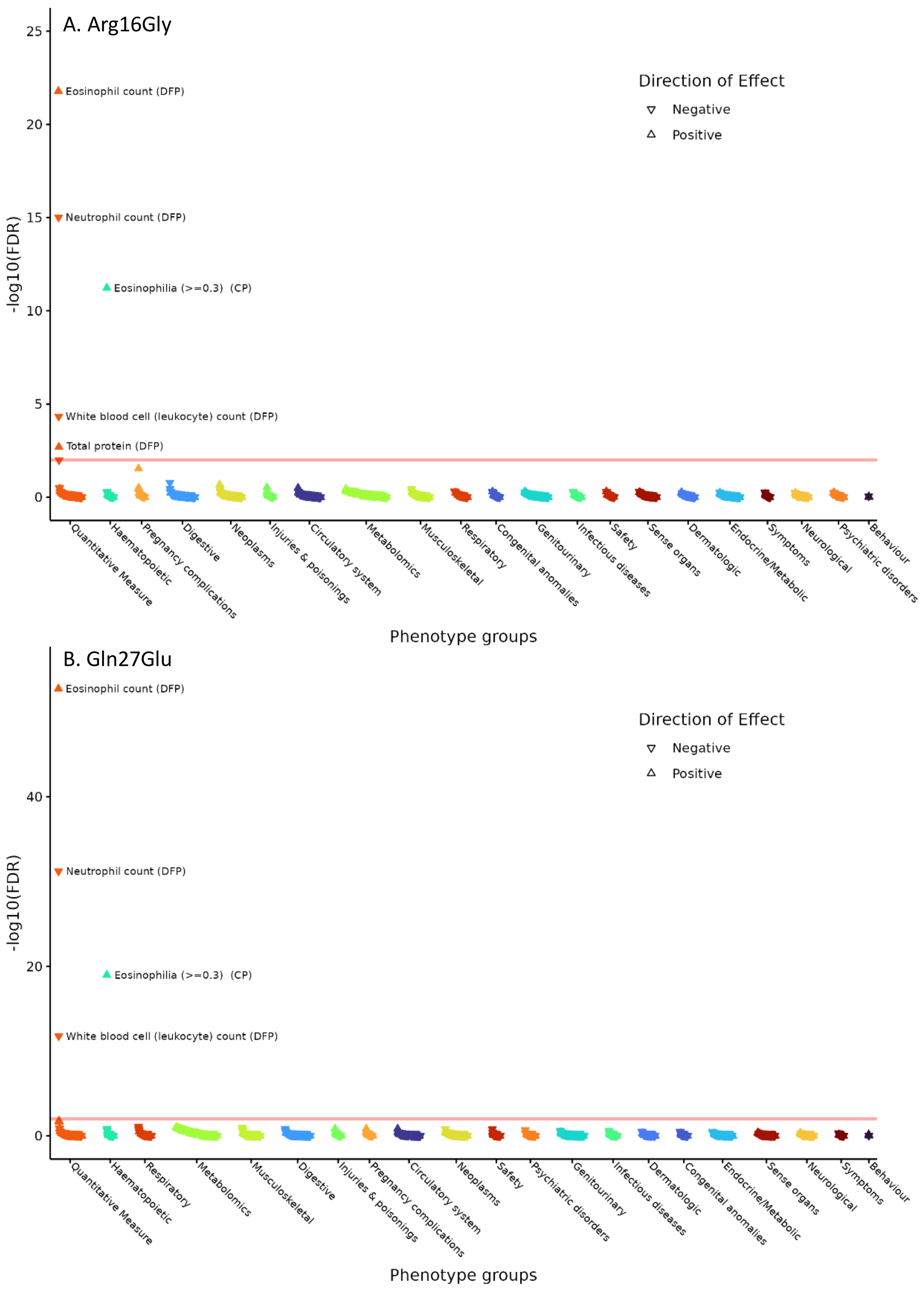

Supplementary Figure 3. Phenome-wide association of UK Biobank traits with Arg16Gly (A) and Gln27Glu (B) polymorphisms. Each triangle is a trait generated by Deep-PheWAS software v0.2.0 and colour-coded according to phenotypic category. The -log10 of the false discovery rate (FDR) is generated from association testing: linear regression (for quantitative traits) or logistic regression (for binary traits), adjusting for age, sex, genotyping array and the first ten ancestry-based principal components (PCs). The red line indicates the false discovery rate threshold of 1%. Directions of effect are aligned to the minor A allele of Arg16Gly and the major C allele of Gln27Glu.

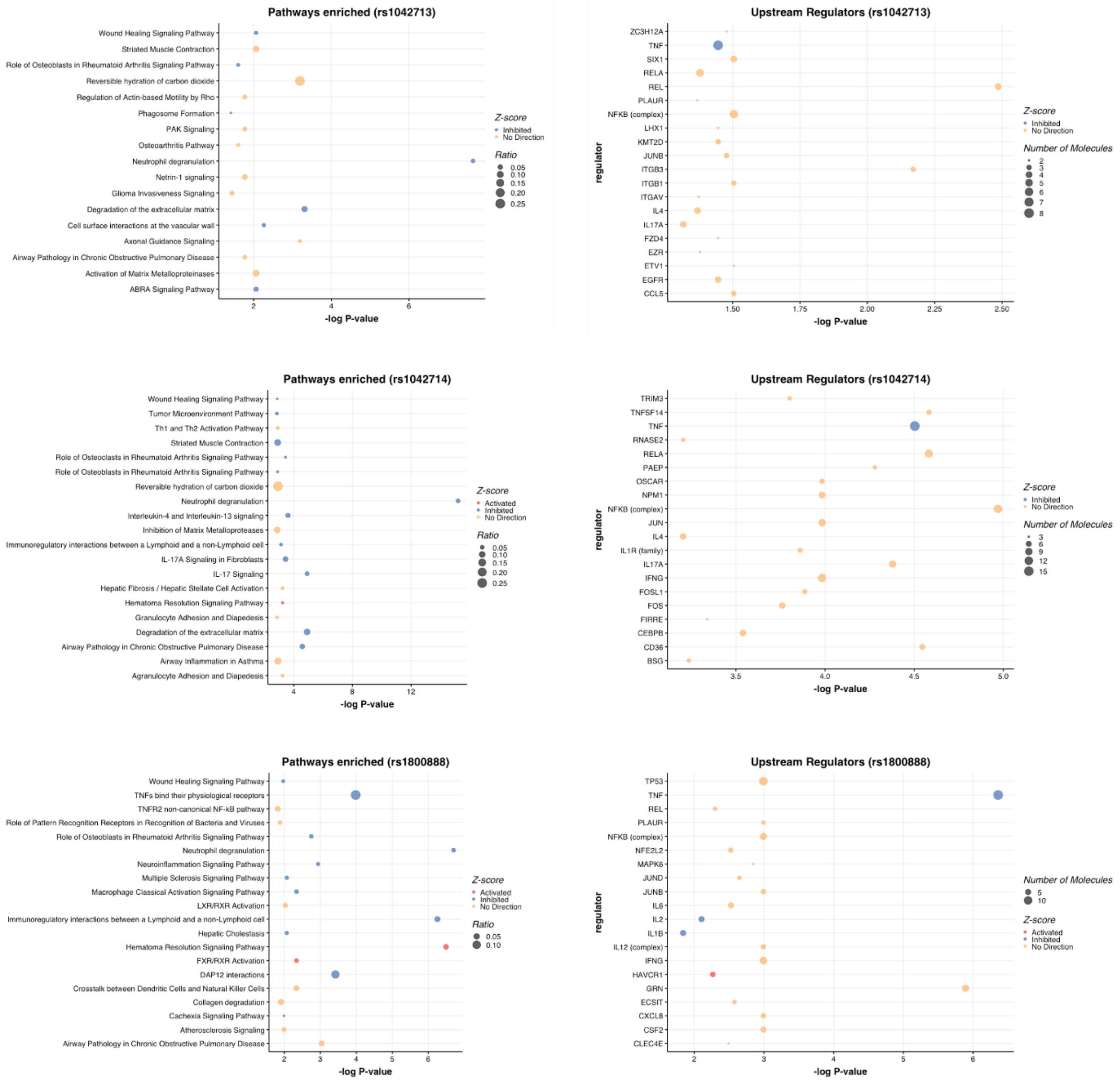

Supplementary Figure 4. Bubble plots of enriched pathways and upstream regulators amongst proteins associated with Arg16Gly (rs1042713), Gln27Glu (rs1042714) and Thr164Ile (rs1800888), generated using Ingenuity Pathway Analysis.

A

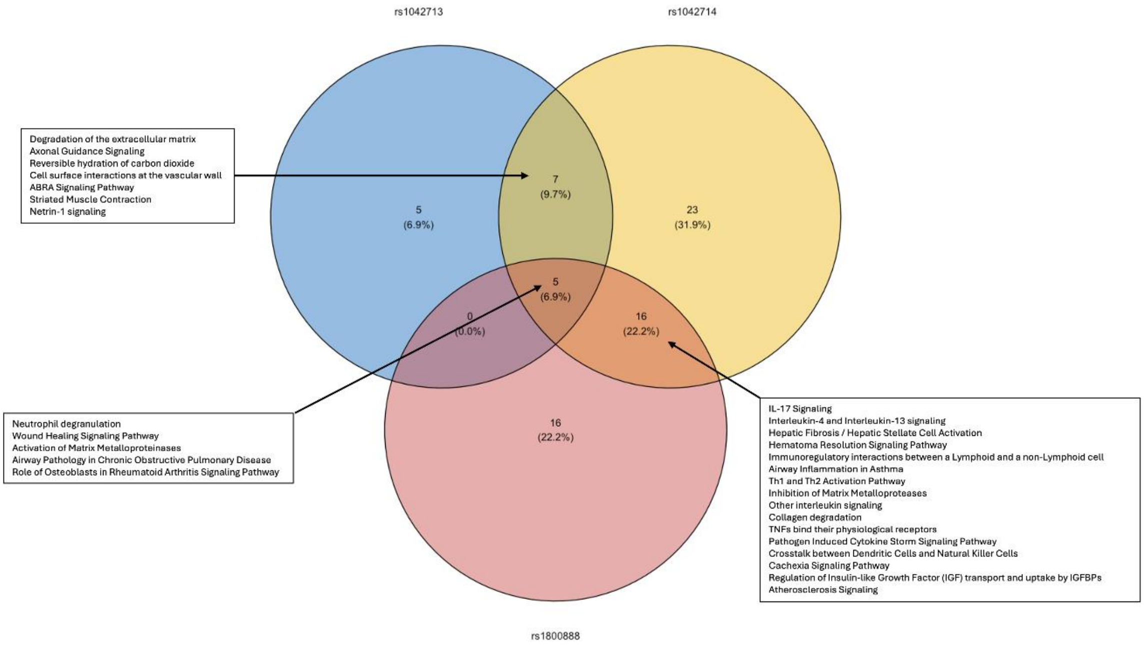

B

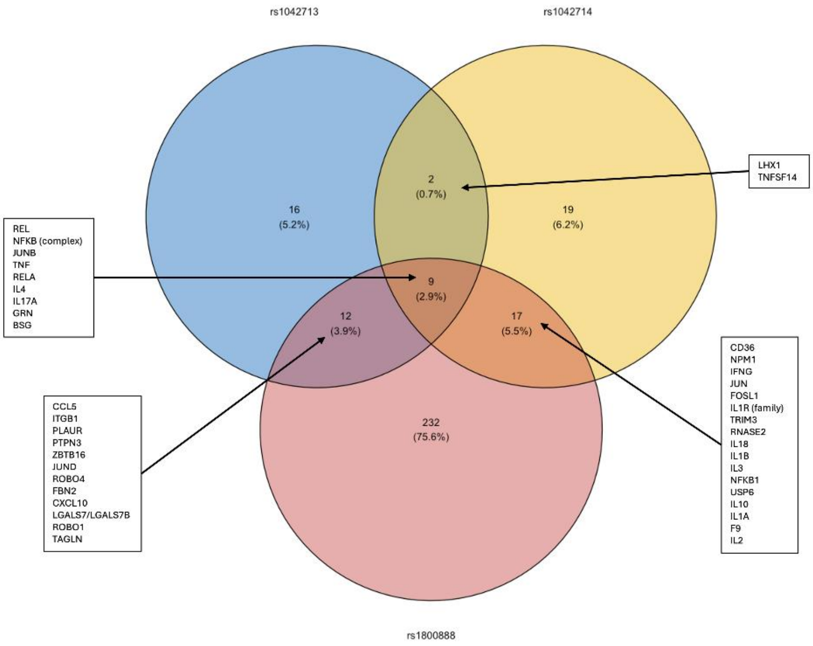

Supplementary Figure 5. Overlap of enriched canonical pathways (A) and upstream regulators (B) amongst proteins associated with Arg16Gly (rs1042713), Gln27Glu (rs1042714) and Thr164Ile (rs1800888).
